# microRNA-27b shuttled by mesenchymal stem cell-derived exosomes prevents sepsis by targeting JMJD3 and downregulating NF-κB signaling pathway

**DOI:** 10.1186/s13287-020-02068-w

**Published:** 2021-01-07

**Authors:** Jia Sun, Xuan Sun, Junhui Chen, Xin Liao, Yixuan He, Jinsong Wang, Rui Chen, Sean Hu, Chen Qiu

**Affiliations:** 1 ShenZhen Beike Biotechnology Research Institute, No. 59, Gaoxin South 9th Road, Nanshan District, Shenzhen, 518057 Guangdong Province People’s Republic of China; 2 Intervention and Cell Therapy Center, Shenzhen Hospital of Peking University, Shenzhen, 518057 People’s Republic of China; 3 Hematology Department, Shenzhen People’s Hospital, Shenzhen, 518020 People’s Republic of China; 4 Clinical Medical Research Center, Shenzhen People’s Hospital, Shenzhen, 518020 People’s Republic of China; 5 Respiratory and Critical Care Medicine Department, Shenzhen People’s Hospital, No. 1017, Dongmen North Road, Luohu District, Shenzhen, 518020 Guangdong Province People’s Republic of China

**Keywords:** Sepsis, Mesenchymal stem cells, Exosome, MicroRNA-27b, Jumonji D3, Nuclear factor κB/p65

## Abstract

**Background:**

Exosomal microRNAs (miRs) derived from mesenchymal stem cells (MSCs) have been shown to play roles in the pathophysiological processes of sepsis. Moreover, miR-27b is highly enriched in MSC-derived exosomes. Herein, we aimed to investigate the potential role and downstream molecular mechanism of exosomal miR-27b in sepsis.

**Methods:**

Inflammation was induced in bone marrow-derived macrophages (BMDMs) by lipopolysaccharide (LPS), and mice were made septic by cecal ligation and puncture (CLP). The expression pattern of miR-27b in MSC-derived exosomes was characterized using RT-qPCR, and its downstream gene was predicted by in silico analysis. The binding affinity between miR-27b, Jumonji D3 (JMJD3), or nuclear factor κB (NF-κB) was characterized to identify the underlying mechanism. We induced miR-27b overexpression or downregulation, along with silencing of JMJD3 or NF-κB to examine their effects on sepsis. The production of pro-inflammatory cytokines TNF-α, IL-1β, and IL-6 was detected by ELISA.

**Results:**

miR-27b was highly expressed in MSC-derived exosomes. Mechanistic investigations showed that miR-27b targeted JMJD3. miR-27b decreased expression of pro-inflammatory genes by inhibiting the recruitment of JMJD3 and NF-κB at gene promoter region. Through this, MSC-derived exosomal miR-27b diminished production of pro-inflammatory cytokines in LPS-treated BMDMs and septic mice, which could be rescued by upregulation of JMJD3 and NF-κB. Besides, in vitro findings were reproduced by in vivo findings.

**Conclusion:**

These data demonstrated that exosomal miR-27b derived from MSCs inhibited the development of sepsis by downregulating JMJD3 and inactivating the NF-κB signaling pathway.

## Background

Sepsis is considered as a global health care problem and remains the leading cause of infectious death [1]. Sepsis is characterized by life-threatening organ dysfunction attributed to a dysregulated widespread activation of host response to infection, which is known as systemic inflammatory response syndrome, leading to large human morbidity and mortality rates each year [2, 3]. Sepsis can trigger complex interactions between the pro-inflammatory and anti-inflammatory processes of the host. A better understanding of inflammatory response-associated mechanisms will provide a new therapeutic approach for the treatment of sepsis [4]. Because therapeutic regimens to ameliorate sepsis are not available, infection is mainly controlled through source control, antibiotics, and organ function support [5]. Thus, identification of biomarkers based on the molecular mechanism underlying sepsis is important to diagnose sepsis, which may also enlighten innovative approaches to treat this disease [6].

Exosomes belong to a class of membrane bound extracellular vesicles that are released by all cells, with a size range from 40 to 150 nm and a composition of bilayer lipid membrane. Exosomes are capable of facilitating multiple intercellular activities, such as communication between cells and activation of signaling pathway [7]. Moreover, it has been reported that exosome-mediated transportation of microRNAs (miRNAs or miRs) may play an important role in sepsis treatment [8].

miRNAs are a class of small, endogenous, non-coding RNAs capable of negatively regulating gene expression by repressing translation or inducing degradation of target mRNA [9]. Dysregulation of miRNAs has been demonstrated to contribute to development and progression of human diseases [10]. Recently, many studies have suggested that abnormally expressed miRNAs play pivotal roles in the initiation and development of sepsis, which suggests that miRNAs may act as potential therapeutic targets of sepsis [11–13]. A previous study reported that exosomes from endothelial progenitor cells confer protection against sepsis by transporting miR-126 [14]. In addition, mesenchymal stem cell (MSC)-derived exosomal miR-223 has been shown to confer cardio-protection in sepsis [15]. More importantly, MSC-based cell therapy showed beneficial effects on sepsis treatment [16]. Several studies have highlighted miR-27b as an anti-inflammatory miRNA in the context of infection [17–19]. Meanwhile, miR-27b has been shown to be significantly decreased in mice with sepsis [20]. Interestingly, miR-27b was found enriched in exosomes isolated from the serum of septic patients [21]. These results suggest that MSC-derived exosomes may play a therapeutic role in sepsis by transferring miR-27b. However, how MSC-derived exosomal miR-27b function in sepsis still remains exclusive.

Based on the bioinformatics analysis and the dual-luciferase reporter gene assay in the present study, miR-27b could target Jumonji D3 (JMJD3), a histone lysine demethylase, regulates transcription and actives the expression of genes via demethylating H3K27me3 [22–24]. More importantly, JMJD3 has been reported to play a critical role in the epigenetic regulation during sepsis [25, 26]. Furthermore, an interaction between JMJD3 and the activation of nuclear factor-κB (NF-κB) has been highlighted [27]. NF-κB participates a typical pro-inflammatory signaling pathway and plays important role in regulating pro-inflammatory gene expression [28], whose dysregulation is linked to many inflammatory diseases including sepsis [29, 30]. Therefore, we hypothesized that MSC-derived exosomal miR-27b could potentially affect the development of sepsis by regulating JMJD3 and NF-κB, which may provide novel therapeutic approaches for the treatment of sepsis.

## Materials and methods

### Ethics statement

The current study was approved by the Ethics Committee of Shenzhen People’s Hospital (ethical approval number: 2020-099) and performed according to the Guide for the Care and Use of Laboratory Animals published by the US National Institutes of Health. Extensive efforts were made to ensure minimal suffering of the animals used in the study.

### Cell culture

Isolation and identification of MSCs: mouse bone marrow-derived mesenchymal stem cells (BMMSCs) were isolated from the tibia and femur bone marrow compartments (*n* = 5) and cultured in a Dulbecco’s modified Eagle’s medium (DMEM) containing 100 μg/mL penicillin and 100 μg/mL streptomycin, 2-mm glutamine, and 15% fetal bovine serum (FBS) in an incubator at 37 °C with 5% CO_2_. The medium was renewed every 3–4 days, and non-adhesive hematopoietic cells were removed. Cells were passaged after treatment with 0.025% trypsin containing 0.02% ethylenediamine tetraacetic acid (EDTA) for 10 min. MSCs at passage 3 were used in this study. Immunofluorescence was applied to identify MSC phenotypes. Briefly, MSCs were incubated with primary anti-mouse antibodies to CD29, Sca-1, and CD34 (1: 100, Affymetrix-eBiosciece, CA, USA), followed by incubation with secondary fluorescent-labeled Alexa Fluor® 488 goat anti-mouse immunoglobulin G (IgG) antibodies (H + L) (1: 500, Gaithersburg, MD, USA). MiR-27b-mimic, negative control (NC)-mimic, miR-27b-inhibitor, NC-inhibitor, small interfering RNA (si)-JMJD3, si-p65, and si-NC (RiboBio, Guangzhou, China) were transfected into MSCs using RNAiMAX (Invitrogen, Carlsbad, CA, USA).

Isolation and culture of bone marrow-derived macrophage (BMDMs): BMDMs were isolated from the bone marrow of 6–8-week-old C57BL/6 male mice (*n* = 5). The femur and tibia were separated with removal of adherent tissues. The two ends of the bone were cut, and the bone marrow was washed with DMEM supplemented with 20% FBS, 100 μg/mL penicillin, 10 μg/mL streptomycin, and 30% L929 cell culture supernatant (containing macrophage and macrophage colony-stimulating factor [M-CSF]), followed by culturing in flask for 7 days. Flow cytometry was used to analyze cell markers CD14 and F4/80 in order to identify BMDMs.

Culture of human embryonic kidney 293T (HEK-293T) cells: HEK-293T cells (CL-0005, Procell, Wuhan, Hubei, China) were cultured in high-glucose DMEM containing 10% FBS, 100 μg/mL penicillin, and 10 μg/mL streptomycin, and L929 cells were cultured in regular DMEM at 37 °C with 5% CO_2._

### Isolation, culture, and differentiation of BMMSCs

Following isolation, BMMSCs were cultured in DMEM-F12 medium (Hyclone Laboratories, Logan, UT, USA) containing 10% FBS (10099141, Gibco, Grand Island, NY, USA), 0.2% penicillin and streptomycin (Hyclone Laboratories, Logan, UT, USA), and sub-cultured every 3 days. The cells at passages 3–7 were used for follow-up experiments. Then, cells were cultured in the medium of osteogenic, adipogenic, or chondrogenic differentiation (all purchased from Cyagen Biosciences Inc., Guangzhou, China) and stained with 0.5% oil red O solution, 5% silver nitrate solution (Von Kossa staining), or 1% alcian blue solution, respectively, to evaluate the accumulation of lipid droplets, calcium deposition, or proteoglycan in cells.

### Flow cytometry for BMMSC identification

BMMSCs at passage 3 with 80% confluence were selected for surface identification. After discarding the culture medium, cells were digested and centrifuged. The pellets were washed twice with phosphate-buffered saline (PBS) buffer, counted with the concentration adjusted to 1 × 10^6^ cells/mL, and transferred into a 15-mL centrifuge tube containing 100 μL of PBS buffer containing 2% FBS. According to the instructions, cells were incubated with specific fluorescent flow cytometric antibodies against CD90, CD105, CD73, CD45, and CD11b (rat anti-mouse, 1:100, labeled by fluorescein isothiocyanate [FITC], BD Biosciences, San Jose, CA, USA) at 4 °C in the dark for 30 min. Thereafter, cells were resuspended with 3 mL of PBS buffer, centrifuged, and added with 300 μL of PBS buffer. In the control group, the background marker was determined by homotype monoclonal antibody, and the fluorescence cells were analyzed by a flow cytometer (BD Biosciences, San Jose, CA, USA). The positive rate of surface antigen (%) was calculated using the FlowJo software (Tree Star, Ashland, OR, USA).

### Establishment of cell and mouse sepsis models

Inflammation was induced in BMDMs (1  ×  10^5^ cells/well) as a cell sepsis model with lipopolysaccharide (LPS; 100 ng/mL, Cat#2630, Sigma-Aldrich Chemical Company, St Louis, MO, USA, purified from *Escherichia coli* [O111: B4]) for 24 h. The supernatant was collected for cytokine measurement to identify the inflammatory model [15].

A sepsis mouse model was established by ligation at 75% of the distal end of the cecum from the base. C57BL/6 male mice (aged 6–8 weeks, weighing 16–22 g) were used for cecal ligation and puncture (CLP). Mice were derived of food for 12 h before operation and then anesthetized by intraperitoneal injection of 2.5% pentobarbital at a dose of 2 mL/kg.

Mice used as control underwent open surgery to separate the distal cecum from mesentery and the abdomen was closed. For sepsis mouse model establishment, the abdomen of mice was routinely disinfected and cut open in the middle to expose the abdominal cavity. Next, the cecum was found, and the mesenteric vessels were separated from the mesenteric vessels carefully to avoid bruising the mesenteric vessels. At the distal end of the cecum, 3/4 was ligated with a sterile No. 4 thread, and a sterile 7-gauge needle was used to pierce the center of the distal end of the occlusal cecum. The cecum was put back into the abdominal cavity, which was closed and sutured layer by layer. After being subjected to CLP, mice were injected 4 h later with MSC-EXO-miR-27b-mimic (30 μg/mouse, exosomes isolated from MSCs transfected with miR-27b-mimic), Ad-overexpression (oe)-JMJD3 (1.5 × 10^9^/mouse, adenovirus expressing oe-JMJD3 plasmids), Ad-oe-p65 (1.5 × 10^9^/mouse, adenovirus expressing oe-p65 plasmids), and Ad-oe-NC (adenovirus expressing oe-NC plasmids). Ad-oe-JMJD3 and Ad-oe-p65 were purchased from Hanbio Biotechnology Co., Ltd. (Shanghai, China) via intravenous tail injection [31, 32]. Tramadol [33] was used to relieve the pain of mice after operation. The mice were divided into two groups: one was used for the survival rate monitor in 7 days, and the other was used for pathological detection. The whole blood and tissues were collected 48 h after CLP treatment for follow-up experiments, with 10 animals in each group.

### Extraction of exosomes

Mouse MSCs were cultured in Roswell Park Memorial Institute 1640 (RPMI-1640) medium containing FBS without exosomes (removal by centrifugation at 100,000 g for 18 h), followed by centrifugation at 2000 g for 10 min and then at 10,000 g for 30 min to remove debris and apoptotic bodies (Avanti-J-26XP, Beckman Coulter, CA, USA). The supernatant was centrifuged at 110,000 g for 70 min (Optima L-80XP, 70 Ti rotor, Beckman Coulter, CA, USA). After centrifugation at 110,000 for 10 min, the precipitate was purified by washing with PBS. All centrifugation was carried out at 4 °C. The pellet was resuspended in PBS and sterilized by filtration through a 0.22-um filter (Millipore, Bedford, MA, USA).

About 10 μL of the exosomal suspension was dropped on the cling film. A 200 mesh copper coating was placed down on the suspension for 60 s. The coating was removed and adsorbed at room temperature for 3 min with the grid facing down. The coating was stained with 2% uranyl acetate for 5 min with the grid facing up. After dried for 5 min, exosome particle size was visualized and quantified under a transmission electron microscopy (TEM) at 80 kV.

Nanoparticle tracking analysis (NTA) was performed to examine exosomes by means of a 405-nm monochromatic laser of the NanoSight NS500 instrument. Exosomes were recorded using NTA software (Malvern Instruments GmbH, Malvern, UK) for 5 times with 30 s at a time. The frame rate was recorded with 25 frames/s. The size of exosome was calculated by Stokes-Einstein equation. Western blot was employed to analyze exosomal markers TAPA-1 (CD81, TA343281, 1: 1000), tumor susceptibility gene 101 (TSG101, TA343598, 1: 500), and syntenin-1 (ABIN1881779, 1: 10000).

### Reverse transcription quantitative polymerase chain reaction (RT-qPCR)

Total RNA from cells and exosomes was extracted with TRIzol reagents (Invitrogen, Carlsbad, CA, USA) using RNeasy Mini Kit (Qiagen, Valencia, CA, USA). Reverse transcription was performed according to the instructions of reverse transcription kit (RR047A, Takara, Japan) to generate complementary DNA (cDNA) as the template for qPCR. cDNA of miRNA was synthesized based on the instructions of First-Strand cDNA Synthesis (Tailing Reaction) kit (B532451-0020, Sangon Biotech Co., Ltd., Shanghai, China). Quantitative PCR was performed with SYBR® Premix Ex Taq™ II (Perfect Real Time) kit (DRR081, Takara, Japan) and processed on qPCR machine (ABI 7500, Foster City, CA, USA). The random negative primer of miRNA and the upstream primer of U6 internal control were provided by miRNA First-Strand cDNA Synthesis (Tailing Reaction) kit. Other primers are listed in Table 1. The relative expression of target genes was quantified by 2^-ΔΔCt^ method normalized to U6. The experiment was repeated 3 times independently.

**Table 1:** Primer sequences for RT-qPCR

Gene	Sequences
miR-27b	F: GGGGTTCACAGTGGCTAA
	R: CAGTGCGTGTCGTGGAGT
U6	F: GCTTCGGCAGCACATATACTAAAAT
	R: CGCTTCACGAATTTGCGTGTCAT

Note: *RT-qPCR* reverse transcription quantitative polymerase chain reaction, *miR-27b* microRNA-27b, *F* forward, *R* reverse

### Enzyme-linked immunosorbent assay (ELISA)

BMDMs were cultured for 48 h after inoculation. The supernatant was collected, centrifuged to remove cells and cell debris, and frozen at − 20 °C to avoid repeated freezing and thawing cycles or directly subjected to detection. The whole blood of the mice was centrifuged at 2000 g for 20 min, and the serum was collected and frozen at − 20 °C to avoid repeated freezing and thawing or directly subjected to detection. The expression of tumor necrosis factor-α (TNF-α; MTA00B), inflammatory cytokine interleukin-1β (IL-1β; MLB00C), and IL-6 (M6000B) proteins in cell culture supernatant and mouse serum was analyzed according to the ELISA kit (R&D System, Minneapolis, MN, USA) instructions. The experiment was repeated 3 times independently.

### Western blot

Total cell proteins were extracted using radio-immunoprecipitation assay (RIPA) lysis buffer (Cat# R0020, Beijing Solarbio Science & Technology Co., Ltd., Beijing, China). Protein concentration was quantified using a bicinchoninic acid (BCA) protein assay kit (Cat# ab102536, Abcam Inc., Cambridge, UK) and adjusted to the same concentration. Proteins were loaded and separated by sodium dodecyl sulfate-polyacrylamide gel electrophoresis (SDS-PAGE). After separation, proteins were transferred to a nitrocellulose membrane, followed by blocking with 5% skim milk for 1–2 h. Diluted primary antibodies JMJD3 (Abcam, Cambridge, UK; 1: 1000), H3K27me3 (Abcam, Cambridge, UK; 1: 2000), H3 (Cell Signaling Technologies, Danvers, MA, USA; 1: 1000), and β-actin (Santa Cruz, CA, USA; 1: 2000) were added and incubated with NC membranes overnight at 37 °C. After washed three times with Tris-buffered saline Tween-20 (TBST), the membranes were incubated with corresponding horseradish peroxidase (HRP)-labeled secondary rabbit anti-mouse IgG (West Grove, PA, USA; 1: 10000) for 2 h. The immunocomplexes on the membrane were visualized using enhanced chemiluminescence (ECL) solution (Thermo Fisher Scientific Inc., Waltham, MA, USA), and band intensities were quantified using ImageJ software. The experiment was repeated 3 times independently.

### Endocytosis of exosomes by BMDMs

Exosomes were pre-labeled with PKH-67 (a new dye for fluorescent labeling of living cells which can be used to label living cells by binding to lipid molecules of membrane structure). PKH-67 has less cytotoxicity to cells, low fluorescence background, high-lipid solubility, and can easily penetrate the cell membrane with strong and stable green fluorescence. PKH-67-labeled cells can be used in vitro and in vivo proliferation studies and have the function of not staining adjacent cells (Sigma-Aldrich Chemical Company, St Louis, MO, USA), followed by centrifugation at 110,000 g for 70 min and washed with PBS to remove the excess staining solution. PKH-67-labeled exosomes (10 mg/mL) were incubated with BMDMs for 24 h. Cells were fixed with 4% paraformaldehyde (PFA) for 10 min and permeabilized with ice cold methanol for 15 min. The nuclei were stained with 4′,6-diamidino-2-phenylindole (DAPI). Images were taken under a laser scanning confocal microscope (LSCM, LSM 510, Carl Zeiss, Germany). Then, the rate of endocytosis was analyzed by flow cytometry.

### Dual-luciferase reporter gene assay

Dual-luciferase reporter gene assay was performed to study the interaction between miR-27b and JMJD3. The potential target genes of miR-27b were predicted via bioinformatics analysis using Starbase (http://starbase.sysu.edu.cn/). The wild-type (wt) 3′untranslated region (UTR) of JMJD3 mRNA sequence containing the predicted target sites of miR-27b was synthesized. The reporter vectors containing JMJD3 wt (pmirGLO-Pygo2-wt) and JMJD3 mutant miR-27b-binding sequence (pmirGLO-JMJD3-mut) or the NC sequence (pmirGLO-NC) (GenePharma, Shanghai, China) were co-transfected into BMDMs with miR-27b-mimic or NC-mimic. After 24-h transfection, activities of firefly luciferase and Renilla luciferase were detected according to the manufacturer’s instruction for the dual-luciferase reporter assay system (Promega, Madison, WI, USA).

### Chromatin immunoprecipitation (ChIP)-qPCR

To study the enrichment of JMJD3 and NF-κB in the promoter region of TNF-α, IL-1β, and IL-6, ChIP was performed using a ChIP assay kit (Millipore, Billerica, MA, USA). Briefly, PBMCs were crosslinked with 1% formaldehyde, washed, and re-suspended in SDS (sodium dodecyl sulfate) lysis buffer. Chromatin was fragmented by sonication. Chromatin fractions were precleared with protein A-agarose beads followed by immunoprecipitation overnight at 4 °C with anti-JMJD3, anti-H3K27me3, anti-NF-κB/p65 antibodies, or with IgG (rabbit IgG-ChIP, ab171870, Abcam, Cambridge, UK) for JMJD3, H3K27me3, and NF-κB; mouse IgG1 (ab81032). All antibodies were obtained from Abcam (Cambridge, UK). Crosslinking was reversed followed by proteinase K incubation. Immunoprecipitated DNA was subjected to qPCR.

### Bacterial colony experiment on sepsis

Solid Luria-Bertani (LB) culture plates were prepared. A serial dilution of 1: 10, 1: 100, 1: 1000, 1: 10,000, and 1: 100,000 was made using peritoneal fluid and serum of mice, respectively. Next, 1 mL of each dilution was uniformly streaked on LB plates, which were incubated at 37 °C for 48 h. Colonies were photographed and the number was counted. The appropriate dilution ratio was selected for subsequent experiments.

### Liver and kidney function determination

Forty-eight hours following CLP, 0.1 mL of fresh whole blood was collected via tail vein [34]. The serum was obtained by centrifugation at 2000 g for 20 min aliquoted and stored at − 20 °C. The levels of liver function indicators, aspartate aminotransferase (AST), and alanine aminotransferase (ALT) and renal function indicator serum creatinine (SCr) were routinely measured using auto-analyzers (Sysmex XT-4000i, Japan; Hitachi 7600-100, Japan; hemagglutination analyzers PUN-2048B, Sysmex, Japan; blood gas analyzer, GEM Premier 3500) [35].

### Hematoxylin-eosin (HE) staining

Forty-eight hours following CLP, the liver, lung, and kidney tissues were collected, fixed with 4% PFA at room temperature for more than 16 h, embedded in paraffin, and sectioned at a 3-um thickness. The sections were immersed in xylene I for 20 min xylene II 20 min, absolute ethanol I 5 min, absolute ethanol II 5 min, and 75% alcohol 5 min to be dewaxed and rehydrated. After rinsing with tap water, the sections were stained with hematoxylin for 3–5 min, blued, dehydrated in increasing concentrations of alcohol (85% and 95%) for 5 min, and counterstained with eosin for 5 min. Thereafter, the sections were cleared with absolute ethanol I for 5 min, absolute ethanol II for 5 min, absolute ethanol III for 5 min, and xylene I and xylene II for 5 min, respectively. The sections were mounted with neutral gum and graphed under a light microscope. At least 10 random fields were examined for each mouse. Lung and kidney injuries were assessed and scored by pathologists blinded to the experiment [14, 31].

### Statistical analysis

The SPSS 21.0 statistical software (IBM Corp., Armonk, NY, USA) was used to analyze statistical data. Measurement data were presented as mean ± standard deviation. Unpaired data in compliance with normal distribution and equal variance between two groups were analyzed using unpaired *t* test. Comparisons among multiple groups were conducted by one-way analysis of variance (ANOVA) with Tukey’s post hoc test. Data at different time points were analyzed by repeated measures ANOVA, followed by Bonferroni post hoc test. Survival curves were calculated using Kaplan-Meier’s method. *p* < 0.05 indicated significant difference.

## Results

### Exosomal miR-27b derived from mouse BMMSCs inhibits CLP-induced sepsis

**Fig. 1:**
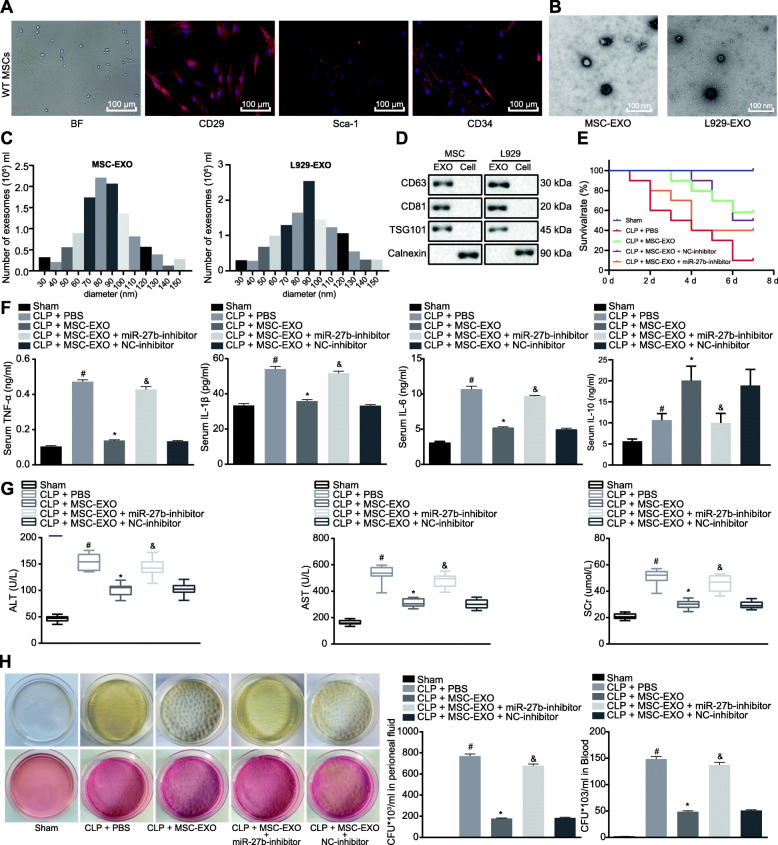
Exosomal miR-27b in mouse BMMSCs inhibits CLP-induced sepsis. **a** Isolation and identification of mouse MSCs, bright field (BF), CD29, Sca-1 and CD34 (green), DAPI (blue). **b** TEM micrographs of mouse MSC-derived exosomes. **c** Size distribution and concentration of particles as quantified by NTA. **d** The expression of miR-27b in mouse MSC-derived exosomes analyzed by RT-qPCR. *p < 0.05 vs. exosomes derived from mouse fibroblasts L929. **e** Measurements of a 7-day survival of mice by Kaplan-Meier method. *n* = 10 for mice following each treatment. **f** The production of TNF-α, IL-1β, IL-10, and IL-6 in serum of CLP-induced septic mice injected with MSC-EXO, MSC-miR-27b-inhibitor-EXO, and MSC-NC-inhibitor-EXO examined by ELISA. **g** Detection of ALT, AST, and SCr levels in the serum of mice. **h** Bacterial colonies in peritoneal fluid and serum of mice. *p < 0.01 vs. CLP-induced mice; #*p* < 0.001 vs. mice with sham operation; &*p* < 0.01 vs. CLP-induced septic mice treated with MSC-miR-27b-inhibitor-EXO. *n* = 10 for mice following each treatment. Quantitative data were presented as mean ± standard deviation. Unpaired data in compliance with normal distribution and equal variance between two groups were compared using unpaired *t* test. Comparisons among multiple groups were analyzed by one-way ANOVA with Tukey’s post hoc test. *p* < 0.05 indicated significant difference

MSC-derived exosomes have protective roles in sepsis [15]. miR-27b is downregulated in the serum sample of sepsis patients [21]. In addition, miR-27b has been found in MSC-derived exosomes [36]. Therefore, it is proposed that exosomal miR-27b may be involved in the development of sepsis. BMMSCs were isolated and cultured, followed by the identification by examining the expression of CD29, Sca-1, and CD34 by immunofluorescence (Fig. 1a). At the same time, we detected MSC surface markers and the potential of MSC differentiation using flow cytometry (Supplementary Figure 1A, B). Exosomes were isolated from the supernatants of mouse MSCs (MSC-EXO) and mouse fibroblasts L929 (L929-EXO) and examined by TEM and NTA (Fig. 1b, c). The expression of miR-27b in MSC-EXO and L929-EXO was quantified by RT-qPCR, and the results of which showed that miR-27b was highly expressed in MSC-EXO compared to L929 control (*p* < 0.05; Fig. 1d). MSC-EXO can inhibit the occurrence of sepsis [15]. The above results showed that miR-27b expression was increased in MSC-EXO, and thus, it was hypothesized that MSCs might be involved with the initiation of sepsis through exosomal miR-27b.

The miR-27b-inhibitor and NC-inhibitor were transfected into BMMSCs for 48 h followed by exosome isolation. RT-qPCR data showed that miR-27b was knocked down in exosomes (Supplementary Figure 2A). The results of 7-day survival rate demonstrated that the survival rate of mice injected with MSC-EXO was higher than that (0%) of CLP-treated mice (*p* < 0.05), while the mice injected with MSC-miR-27b-inhibitor-EXO showed decreased survival rate compared with those injected with MSC-NC-inhibitor-EXO (*p* < 0.05; Fig. 1e). Furthermore, as detected by ELISA, the production of TNF-α, IL-1β, IL-10, and IL-6 was increased in the serum of CLP-induced mice, compared to sham-operated mice. However, TNF-α, IL-1β, and IL-6 presented a decreased production and IL-10 production was increased in response to treatment with MSC-EXO. Mice injected with MSC-miR-27b-inhibitor-EXO had higher expression of TNF-α, IL-1β, and IL-6, yet lower IL-10 expression than that with MSC-EXO (*p* < 0.05; Fig. 1f). Additionally, the levels of ALT, AST, and SCr were lower in MSC-EXO-treated CLP-induced mice than that in PBS-treated CLP-induced mice (*p* < 0.05), which was reversed when comparing MSC-miR-27b-inhibitor-EXO-treated mice with SC-EXO-treated mice (*p* < 0.05; Fig. 1g).

The number of colonies was smaller in peritoneal fluid and serum of CLP-induced mice treated with MSC-EXO than that in mice treated with PBS (*p* < 0.01), but the number of colonies in mice injected with MSC-miR-27b-inhibitor-EXO was larger than that in mice injected with MSC-EXO (*p* < 0.01; Fig. 1h).

The liver, kidney, and lung tissue sections of mice were observed with HE staining. The results revealed that the liver, kidney, and lung injury scores of MSC-EXO-treated CLP-induced mice were decreased. Compared with MSC-NC-inhibitor-EXO-treated mice, the injury scores of MSC-miR-27b-inhibitor-EXO-treated mice were increased (*p* < 0.01; Supplementary Figure 2B). Taken together, knockdown of miR-27b could reverse the inhibitory effect of MSC-EXO on sepsis.

### miR-27b attenuates LPS-mediated BMDM inflammation by inhibiting JMJD3

**Fig. 2:**
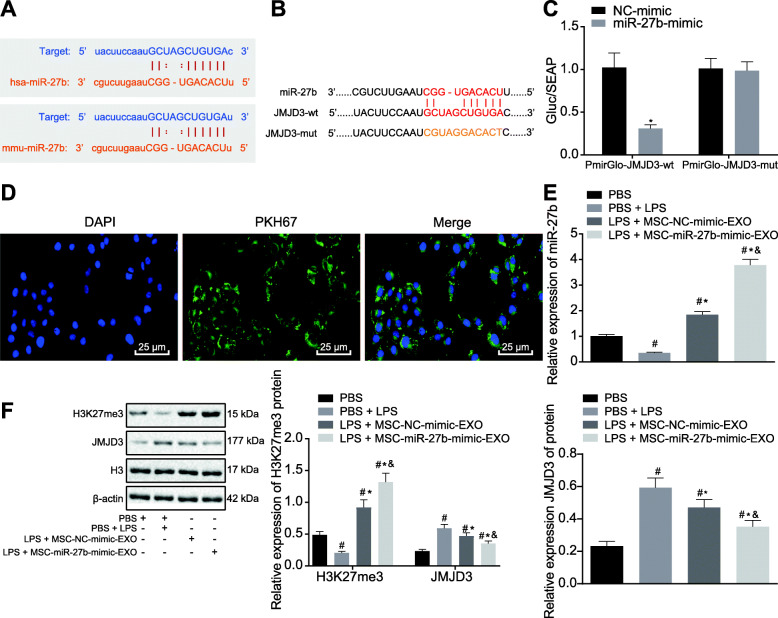
miR-27b prevents LPS-mediated BMDM inflammation through suppression of JMJD3. **a** Target genes of miR-27b predicted by the Starbase website. **b**, **c** Binding sites (**b**) of miR-27b to JMJD3 and verification (**c**) by dual-luciferase reporter gene assay. *p < 0.01 vs. BMDMs transfected with NC-mimic*.* **d** Images of BMDMs uptaking exosomes visualized under a LSCM. **e** The expression of miR-27b in BMDMs treated with LPS or MSC-NC-mimic-EXO and MSC-miR-27b-mimic-EXO analyzed by RT-qPCR. **f** The expression of JMJD3 and H3K27me3 in BMDMs treated with LPS or MSC-NC-mimic-EXO and MSC-miR-27b-mimic-EXO analyzed by western blot. In panel 2 **e**, **f**, *p < 0.05 vs. PBS-treated BMDMs; #*p* < 0.05 vs. LPS-treated BMDMs; &*p* < 0.05 vs. BMDMs treated with MSC-NC-mimic-EXO. The experiment was repeated 3 times independently. Quantitative data were presented as mean ± standard deviation. Unpaired data in compliance with normal distribution and equal variance between two groups were compared using unpaired *t* test. Comparisons among multiple groups were analyzed by one-way ANOVA with Tukey’s post hoc test. *p* < 0.05 indicated significant difference

The molecular mechanism of how miR-27b inhibits sepsis was explored. The target genes of miR-27b were predicted using the Starbase database, which revealed that miR-27b targeted the JMJD3 mRNA 3′UTR in both mice and humans (Fig. 2a). Subsequently, dual-luciferase reporter gene assay was applied to verify their interaction. pmirGLO-JMJD3-wt and pmirGLO-JMJD3-mut plasmid were constructed (Fig. 2b), which were co-transfected with miR-27b-mimic into the BMDMs. The results displayed that miR-27b increased the activity of luciferase in BMDMs transfected with JMJD3-wt (*p* < 0.01; Fig. 2c).

To verify whether exosomes derived from MSCs could be taken up by BMDMs, PKH-67-labeled MSCs and unlabeled exosomes were incubated with BMDMs for 24 h. Images were taken under a LSCM (Fig. 2d), and the endocytosis of exosomes by BMDMs was found to be more than 90%. BMDMs were treated with LPS (100 ng/mL) to establish a sepsis model. miR-27b-mimic was transfected into MSCs, and MSC-derived exosomes (MSC-miR-27b-mimic-EXO) were isolated. RT-qPCR was used to analyze miR-27b expression in BMDMs, the results of which showed that the expression of miR-27b in BMDMs was reduced under LPS treatment compared to control group, which was rescued by the treatment of MSC-miR-27b-mimic-EXO (*p* < 0.05; Fig. 2e).

The expression of JMJD3 and H3K27me3 in BMDMs was analyzed by Western blot. Results showed that the expression of JMJD3 was increased while that of H3K27me3 was decreased in the LPS-treated BMDMs in comparison with PBS-treated BMDMs (*p* < 0.05), which was abolished by MSC-NC-mimic-EXO or MSC-miR-27b-mimic-EXO treatment, as evidence by reduced expression of JMJD3 (*p* < 0.05) and increased H3K27me3 expression (*p* < 0.01) (Fig. 2f).

ELISA assay was applied to examine the expression of inflammatory cytokines including TNF-α, IL-1β, and IL-6 and anti-inflammatory factor IL-10 in the supernatant of BMDMs. Expression of TNF-α, IL-1β, and IL-6 induced by LPS was downregulated while that of IL-10 was increased in the presence of MSC-miR-27b-mimic-EXO (*p* < 0.05) (Supplementary Figure 3A).

Whether the therapeutic effects of MSC-EXO on LPS-induced inflammation as cell sepsis model were associated with miR-27b was further investigated. miR-27b-inhibitor was transfected into MSCs and exosomes (MSC-miR-27b-inhibitor-EXO) were isolated. MSC-miR-27b-inhibitor-EXO was co-incubated with LPS-treated BMDMs. ELISA was used to determine the expression of TNF-α, IL-1β, IL-6, and IL-10 in cell supernatant. The results demonstrated that inhibition of miR-27b repressed the effect of MSC-EXO on inflammation (Supplementary Figure 3B). In summary, high expression of miR-27b in MSC-derived exosomes could inhibit the expression and function of JMJD3 on LPS-induced inflammation leading to the repression of sepsis.

### JMJD3 interacting with NF-κB/p65 increases expression of pro-inflammatory cytokines in LPS-induced BMDMs

The aforementioned results demonstrated that miR-27b targeted and downregulated JMJD3 to decrease the production of pro-inflammatory cytokines in BMDMs so as to inhibit the inflammation in sepsis. So, how JMJD3 regulated the expression of pro-inflammatory cytokines was further investigated. JMJD3 is known as a demethylase that regulates the transcription of target genes, and meanwhile, it has been shown to coordinate with NF-κB/p65 to regulate transcription of target genes [27]. Moreover, NF-κB/p65 is involved in the initiation of sepsis [37]. Therefore, we wanted to study whether JMJD3 and NF-κB/p65 co-regulated the expression of pro-inflammatory cytokines.

**Fig. 3:**
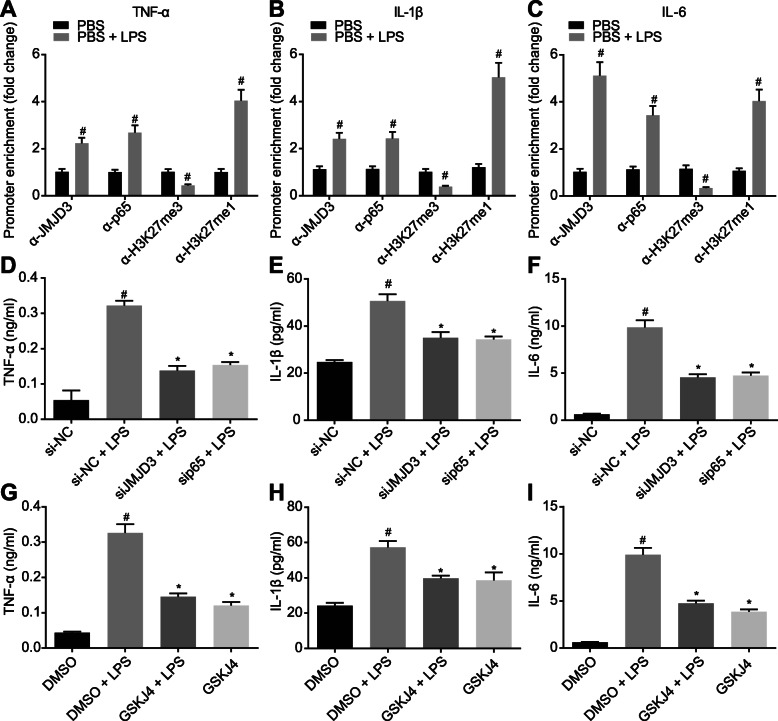
JMJD3 interacting with NF-κB/p65 elevates expression of the pro-inflammatory cytokines in LPS-induced BMDMs. **a–c** The recruitment of JMJD3, p65, H3K27me3, and H3K27me1 in the promoter region of pro-inflammatory cytokines TNF-α (**a**), IL-1β (**b**), and IL-6 (**c**) in BMDMs analyzed by CHIP-qPCR. #*p* < 0.05 vs. PBS-treated BMDMs. **d**–**f** The expression of LPS-induced pro-inflammatory factors TNF-α (**d**), IL-1β (**e**), and IL-6 (**f**) after knockdown of JMJD3 and p65. *p < 0.05 vs. PBS-treated BMDMs in response to si-NC; #*p* < 0.05 vs. BMDMs in response to si-NC. **g**–**i** The expression of pro-inflammatory factors TNF-α (**g**), IL-1β (**h**), and IL-6 (**i**) in BMDMs after 1-h treatment with GSK-J4 (4 umol/L, pharmacological inhibitor of JMJD3). *p < 0.05 vs. LPS-treated BMDMs in response to GSK-J4; #*p* < 0.05 vs. BMDMs in response to GSK-J4. The experiment was repeated 3 times independently. Quantitative data were presented as mean ± standard deviation. Unpaired data in compliance with normal distribution and equal variance between two groups were compared using unpaired *t* test. Comparisons among multiple groups were analyzed by one-way ANOVA with Tukey’s post hoc test. *p* < 0.05 indicated significant difference

The recruitment of JMJD3 and NF-κB/p65 in the promoter region of pro-inflammatory factor was analyzed by CHIP-qPCR. The promoter region of pro-inflammatory cytokines TNF-α, IL-1β, and IL-6 in LPS-treated BMDMs showed increased enrichment of JMJD3 and NF-κB/p65, decreased H3K27me3, increased H3K27me1, and upregulated expression of pro-inflammatory cytokines (*p* < 0.05; Fig. 3a–c).

To further validate the effects of JMJD3 and p65 on the expression of pro-inflammatory cytokines, LPS-induced pro-inflammatory factor expression was inhibited after knockdown of JMJD3 and p65 by siRNA (*p* < 0.05; Fig. 3d–f). After treatment with GSK-J4 (4 μmol/L, ab144395, Abcam Inc., Cambridge, UK), a pharmacological inhibitor of histone demethylase JMJD3, inflammatory response of LPS-induced BMDMs was reduced (*p* < 0.05; Fig. 3g–i). These results indicated that LPS induced the recruitment of JMJD3 and NF-κB in the promoter region of pro-inflammatory cytokines and H3K27me3 demethylation, leading to the regulation of gene transcription.

### MSC-EXO suppresses LPS-induced inflammation in BMDMs through regulating miR-27b and inhibiting JMJD3 and NF-κB/p65

**Fig. 4:**
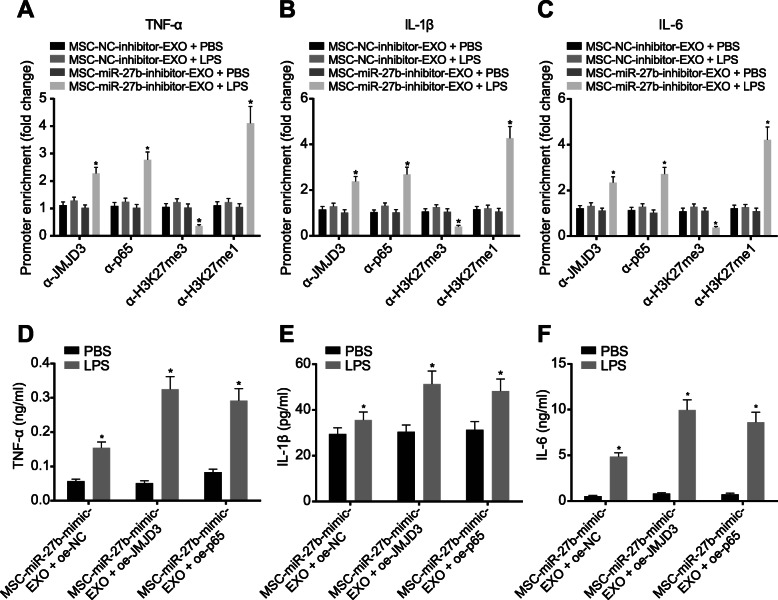
MSC-derived exosomal miR-27b inhibits JMJD3 and NF-κB/p65 axis to restrain LPS-induced pro-inflammatory response. **a–c** The recruitment of JMJD3, p65, H3K27me3, and H3K27me1 in the promoter region of pro-inflammatory cytokines TNF-α (**a**), IL-1β (**b**), and IL-6 (**c**) in LPS-treated BMDMs transfected with MSC-miR-27b-inhibitor-EXO analyzed by CHIP-qPCR. *p < 0.05 vs. LPS-treated BMDMs transfected with MSC-NC-inhibitor-EXO. **d**–**f** The expression of pro-inflammatory cytokines TNF-α (**d**), IL-1β (**e**), and IL-6 (**f**) in BMDMs after overexpression of JMJD3 or p65. *p < 0.05 vs. PBS-treated BMDMs. The experiment was repeated 3 times independently. Quantitative data were presented as mean ± standard deviation. Unpaired data in compliance with normal distribution and equal variance between two groups were compared using unpaired *t* test. Comparisons among multiple groups were analyzed by one-way ANOVA with Tukey’s post hoc test. *p* < 0.05 indicated significant difference

The molecular mechanism of anti-inflammatory effect of MSC-EXO on LPS-induced BMDMs was further investigated. The inhibitory effect of miR-27b in MSC-EXO on the recruitment of JMJD3 and NF-κB/p65 in the pro-inflammatory cytokine promoter regions in LPS-induced BMDMs was analyzed by ChIP-qPCR. The MSCs were transfected with miR-27b-inhibitor and NC-inhibitor for 48 h, after which the exosomes were isolated and the exosomes with low expression of miR-27b were incubated with LPS-treated BMDMs. ChIP-qPCR was then performed to detect the recruitment of JMJD3 and NF-κB/p65 in the pro-inflammatory cytokine promoter regions, and the results of which showed that knockdown of miR-27b reversed the inhibitory effect of MSC-EXO on LPS-mediated BMDM inflammatory response (*p* < 0.05). Meantime, knockdown of miR-27b obviously changed the expression of H3K27 in the promoter region of inflammatory factors (*p* < 0.05; Fig. 4a–c). Overexpression of JMJD3 and p65 also inhibited the effect of MSC-EXO on LPS-mediated inflammation of BMDMs (*p* < 0.05; Fig. 4d–f). In summary, MSC-derived exosomal miR-27b inhibited LPS-induced pro-inflammatory effects on BMDMs by inhibiting JMJD3 and NF-κB/p65 axis.

### MSC-derived exosomal miR-27b attenuates CLP-induced sepsis in mice via inhibition of JMJD3/NF-κB/p65 axis

**Fig. 5:**
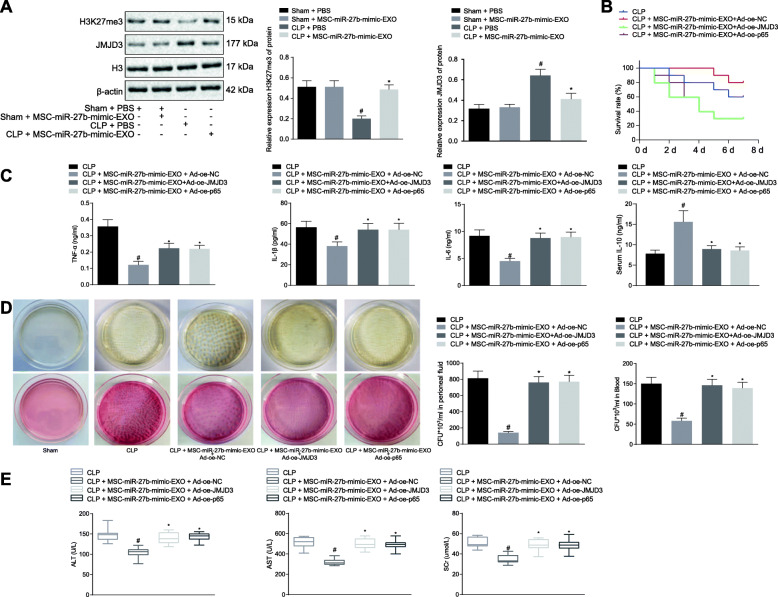
Exosomal miR-27b from MSCs suppresses the JMJD3/NF-κB/p65 axis to inhibit the development of CLP-induced sepsis. **a** The expression of JMJD3 and H3K27me3 in mice subjected to sham operation or CLP in response to MSC-miR-27b-mimic-EXO analyzed by Western blot. *p < 0.05 vs. mice subjected to CLP; #*p* < 0.05 vs. mice subjected to sham operation. **b** Results of a 7-day survival in mice. *n* = 10 for mice following each treatment. **c** The production level of TNF-α, IL-1β, IL-10, and IL-6 in mouse serum examined by ELISA. **d** Bacterial colonies in peritoneal fluid and serum of mice. **e** Detection of the liver and kidney function in mice. *p < 0.05 vs. mice subjected to CLP. *n* = 10 for mice following each treatment. Cellular experiments were repeated 3 times independently. Quantitative data were presented as mean ± standard deviation. Comparisons among multiple groups were analyzed by one-way ANOVA with Tukey’s post hoc test. *p* < 0.05 indicated significant difference

Mice subjected to CLP could simulate sepsis, which was utilized to validate our results in vivo. Western blot was used to measure expression of JMJD3 and H3K27me3 in BMDMs of CLP-treated mice or CLP-treated mice in response to MSC-miR-27b-mimic-EXO. The results revealed that CLP-treated mice in response to MSC-miR-27b-mimic-EXO showed a significant increase in JMJD3 expression but a decrease in H3K27me3 expression (*p* < 0.05; Fig. 5a). Analysis of 7 days of survival curve demonstrated that the survival rate of the mice was decreased in the presence of MSC-miR-27b-mimic-EXO and upregulation of JMJD3 and p65 (*p* < 0.05; Fig. 5b).

Ad-oe-JMJD3 and Ad-oe-p65 were injected into CLP-treated mice. The production of pro-inflammatory cytokines TNF-α, IL-1β, IL-10, and IL-6 in serum was detected by ELISA, which demonstrated that upregulation of JMJD3 and p65 inhibited effect of MSC-miR-27b-mimic-EXO on inflammation in CLP-treated mice (*p* < 0.05; Fig. 5c).

The number of colonies in the peritoneal fluid and blood of mice was quantified, which was found to be increased after overexpression of JMJD3 and p65 (*p* < 0.05; Fig. 5d).

Analysis of the liver and kidney function showed overexpressing of JMJD3 and p65 promoted the ALT, AST, and SCr indexes and indicated liver and kidney dysfunction (*p* < 0.05; Fig. 5e). HE staining showed that the liver, lung, and kidney tissue injury scores were increased and tissue injury was worse after overexpression of JMJD3 and p65 (*p* < 0.05; Supplementary Figure 4). In summary, the CLP mouse model confirmed that overexpression of miR-27b in MSC-EXO could suppress the JMJD3/NF-κB/p65 axis to inhibit the development of sepsis in vivo.

## Discussion

Sepsis is a syndrome attributed to infections of body immune and coagulation systems leading to approximately 44,000 deaths each year in UK, which is likely to cause shock, multiple organ failure, and even death without early detection and proper treatment [38]. Currently, no specific treatment to sepsis is available [5]. Thus, novel therapies tailored with the aid of molecular biomarkers are urgently needed to diagnose and prevent sepsis. MSCs have been reported to improve the survival of Gram-negative diabetic peritoneal sepsis [39–41]. However, the specific mechanism of how MSCs function in sepsis remains poorly understood. Our data demonstrated that MSCs were able to ameliorate sepsis via the exosome-mediated transportation of miR-27b through regulating the JMJD3/NF-κB/p65 axis.

In fact, the therapeutic and regenerative effects of MSCs may not only depend on local mechanisms but through the secretion of paracrine cytokines such as exosomes [42, 43]. MSCs could exert anti-inflammatory effects through the exosome-mediated transportation of miR-223 in polymicrobial sepsis [15]. In addition, the anti-inflammatory effect of MSC-derived exosomes was also presented in sepsis by carrying miR-146a [31]. miR-27b has been demonstrated to be poorly expressed in the serum of patients with sepsis [21] but highly expressed in MSC-derived exosomes. The present data revealed that exosomal miR-27b from MSCs showed an inhibitory effect on the development of sepsis as evidenced by decreased injuries in the liver, kidney, and lung in CLP-treated mice.

In a mouse model of sepsis, miR-27a was found to be significantly decreased and its overexpression can alleviate inflammatory response, thus preventing the LPS-induced liver injury following sepsis [20]. Bioinformatics analysis and dual-luciferase reporter gene assay together identified that miR-27b targeted JMJD3 and downregulated its expression. This contributed to the reduction of LPS-mediated BMDM inflammation, accompanied by decreased expression of LPS-stimulated inflammatory cytokines TNF-α, IL-1β, and IL-6 as well as increased expression of anti-inflammatory factor IL-10. Partially in agreement with our results, miR-939 has been reported to target and downregulate JMJD3 to inhibit the multiplication of hepatitis B virus [44]. Additionally, miR-146a could suppress osteogenesis in MSCs by downregulating JMJD3 expression [45]. Some papers have shown that JMJD3 can facilitate great effects on several inflammatory diseases including atherosclerosis [46], rheumatoid arthritis [47], and sepsis [48]. JMJD3 was demonstrated to be upregulated in cells treated with LPS, along with a reduced expression of H3K27me3- and LPS-mediated inflammation [26]. The immune system responds by secreted inflammatory molecules, whose dysregulation results in chronic inflammation disease and tissue damage [49]. Pro-inflammatory cytokines, including TNF-α, IL-1β, and IL-6 play an important role in the development of chronic inflammation [50, 51]. JMJD3 demonstrates promoting properties in sepsis by upregulating pro-inflammatory cytokines, IL-1β, and TNF-α expression [51]. Furthermore, miR-27-3p has the capacity to increase the production of IL-10 in dendritic cells [52]. Taken together, we concluded that exosomal miR-27b derived from MSCs could target and downregulate JMJD3 to reduce the inflammatory responses in sepsis.

JMJD3 is known as a H3K27 demethylase, which regulates the transcription of target genes through H3K27me3 demethylation [53]. A previous study showed that JMJD3 synergistically regulated the transcription of target genes with the transcription regulator NF-κB/p65 [27] and emerging evidences demonstrated that NF-κB/p65 was associated with the development of sepsis [54, 55]. More importantly, suppression of NF-κB contributed to a reduction of release of LPS-induced pro-inflammatory cytokines such as TNF-α, IL-1β, and IL-6 in sepsis [37, 56]. Furthermore, a previous study highlighted the ability of miR-27b to suppress the activity of NF-κB, thus arresting an excessive inflammation during infection [18]. In consistent with these results, we demonstrated that the recruitment of JMJD3 and NF-κB in the promoter regions of TNF-α, IL-1β, and IL-6 contributed to the upregulation of these pro-inflammatory cytokines via H3K27me3 demethylation, thus promoting inflammatory response in sepsis. Our work also revealed that MSC-derived exosomes through the transportation of miR-27b regulated JMJD3/NF-κB axis to inhibit LPS-induced BMDM pro-inflammatory response as well as CLP-induced sepsis in mice.

## Conclusions

**Fig. 6:**
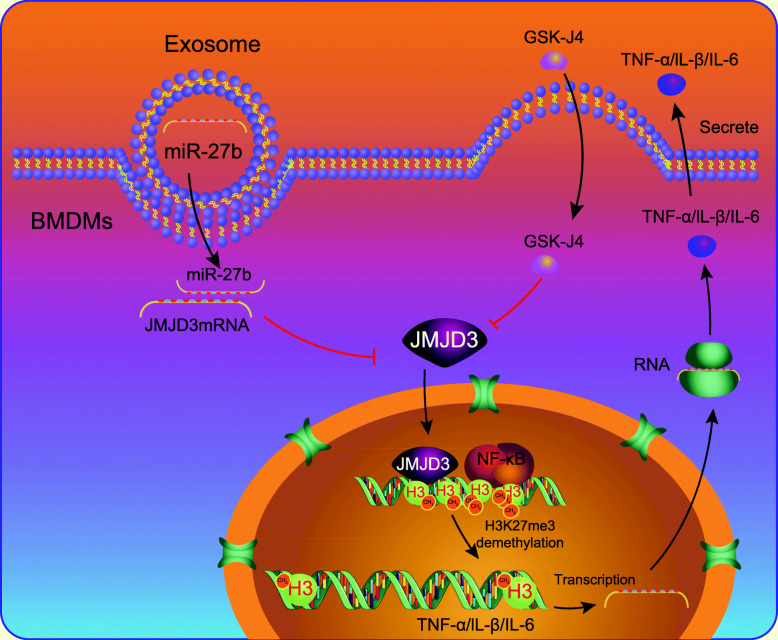
Schematic diagram of the function and mechanism of exosomal miR-27b in sepsis. JMJD3 can be transferred into nucleus through cytoplasm and nucleus and promote the demethylation of H3K27me3 by recruiting NF-κB, thus activating the transcription of TNF-α/IL-β/IL-6 to increase the expression of these target proteins. However, miR-27b loaded in the exosomes secreted by MSCs binds to the mRNA of JMJD3 in the target cells after membrane fusion and inhibits the expression of JMJD3 protein, thus suppressing the expression of inflammatory factors. GSK-J4, a pharmacological inhibitor of JMJD3, has similar effects

In summary, miR-27b delivered by MSC-derived exosomes could downregulate JMJD3 and NF-κB/p65 to inhibit inflammatory response and suppress sepsis (Fig. 6). Therefore, investigation of the miR-27b/JMJD3/NF-κB/p65 axis and their functions presents new mechanistic insights for an understanding of sepsis initiation and development and provides potential novel therapeutic targets against sepsis. Nevertheless, in addition to JMJD3, there are many other genes that are targeted by miR-27b [18, 19] and the specific mechanism involving the miR-27b/JMJD3 axis thus needs to be further identified through additional studies. Additionally, it would be necessary for future studies to investigate specific mechanistic basis by which overexpression of miR-27b as well as inhibition of JMJD3 and NF-κB/p65 could impede the progression of sepsis.

## Supplementary Information

**Additional file 1: Supplementary Figure 1.** Characterization of BMMSCs. A, adipogenic (the left panel), osteogenic (the middle panel) and chondrogenic (the right panel) differentiation of MSCs. B, MSC surface makers CD105, CD73, CD90, CD45 and D11b analyzed by flow cytometry.

**Additional file 2: Supplementary Figure 2.** Inhibition of miR-27b reverses the inhibitory effect of MSC-EXO on CLP-induced liver, kidney and lung injuries. .A, miR-27b expression determined by RT-qPCR in exosomes derived from MSCs transfected with miR-27b inhibitor. B, HE staining of liver, kidney and lung tissues of mice treated with MSC-EXO or MSC-miR-27b-inhibitor-EXO (400 ×). *, *p* < 0.01 vs. CLP-induced septic mice; #, *p* < 0.001 vs. mice with sham operations; &, *p* < 0.01 vs. CLP-induced septic mice treated with MSC-NC-inhibitor-EXO. *n* = 10 for mice following each treatment. Quantitative data were presented as mean ± standard deviation. Comparisons among multiple groups were analyzed by one-way ANOVA with Tukey’s post hoc test. *p* < 0.05 indicated significant difference.

**Additional file 3: Supplementary Figure 3.** Inhibition of miR-27b represses the effect of MSC-EXO on LPS-induced inflammation. A, The expression levels of inflammatory cytokines TNF-α, IL-1β, IL-10 and IL-6 in the supernatant of BMDMs in response to LPS, MSC-NC-mimic-EXO and MSC-miR-27b-mimic-EXO measured by ELISA assay. *, *p* < 0.05 vs. PBS-treated BMDMs; #, *p* < 0.05 vs. LPS-treated BMDMs; &, *p* < 0.05 vs. BMDMs treated with MSC-NC-mimic-EXO. B, The expression of inflammatory cytokines TNF-α, IL-1β, IL-6 and IL-10 in the supernatant of BMDMs treated with MSCs-miR-27b-inhibitor-EXO. #, *p* < 0.05 vs. LPS-treated BMDMs; &, *p* < 0.05 vs. BMDMs treated with MSC-NC-mimic-EXO. The experiment was repeated 3 times independently. Quantitative data were presented as mean ± standard deviation. Comparisons among multiple groups were analyzed by one-way ANOVA with Tukey’s post hoc test. *p* < 0.05 indicated significant difference.

**Additional file 4: Supplementary Figure 4**. HE staining of liver, kidney and lung tissues of mice treated with MSC-miR-27b-mimic-EXO, MSC-miR-27b-mimic-EXO + Ad-oe-p65 or MSC-miR-27b-mimic-EXO + Ad-oe-JMJD3 (400 ×). *, *p* < 0.05 vs. mice subjected to CLP. n = 10 for mice following each treatment. Quantitative data were presented as mean ± standard deviation. Comparisons among multiple groups were analyzed by one-way ANOVA with Tukey’s post hoc test. *p* < 0.05 indicated significant difference.

## Data Availability

The datasets generated/analyzed during the current study are available.

## Abbreviations

MSCs: Mesenchymal stem cells
miRNAs or miRs: MicroRNAs
miR-27b: MicroRNA-27b
BMMSCs: Bone marrow-derived mesenchymal stem cells
BMDMs: Bone marrow macrophages
MSC-EXO: MSC-derived exosomes
CLP: Cecal ligation and puncture
NF-κB: Nuclear factor κB
JMJD3: Jumonji D3
DMEM: Dulbecco’s modified Eagle’s medium
HEK-293T: Human embryonic kidney 293T
TEM: Transmission electron microscopy
NTA: Nanoparticle tracking analysis
LSCM: Laser scanning confocal microscope
PFA: Paraformaldehyde
FBS: Fetal bovine serum
oe: Overexpression
siRNA: Small interfering RNA
NC: Negative control
EDTA: Ethylenediaminetetraacetic acid
LPS: Lipopolysaccharide
3′UTR: 3′untranslated region
PBS: Phosphate-buffered saline
IL-6: Interleukin-6
TNF-α: Tumor necrosis factor-α
FITC: Fluorescein isothiocyanate
cDNA: Complementary DNA
RT-qPCR: Reverse transcription quantitative polymerase chain reaction
RIPA: Radio-immunoprecipitation assay
BCA: Bicinchoninic acid
SDA-PAGE: Sodium dodecyl sulfate polyacrylamide gel electrophoresis
IgG: Immunoglobulin G
TBST: Tris-buffered saline with Tween-20
HRP: Horseradish peroxidase
ECL: Enhanced chemiluminescence
ChIP: Chromatin immunoprecipitation
AST: Aspartate aminotransferase
ALT: Aminotransferase
SCr: Serum creatinine
HE: Hematoxylin and eosin
DAPI: 4′,6-Diamidino-2-phenylindole
wt: Wild type
mut: Mutant
ELISA: Enzyme-linked immunosorbent assay
ANOVA: Analysis of variance
